# The Effect of Long-Term (Im)balance of Giving Versus Receiving Support With Nonrelatives on Subjective Well-Being Among Home-Dwelling Older People

**DOI:** 10.1093/geronb/gbad198

**Published:** 2024-01-03

**Authors:** Wenran Xia, Jeroen D H van Wijngaarden, Robbert Huijsman, Martina Buljac-Samardžić

**Affiliations:** Erasmus School of Health Policy and Management, Erasmus University Rotterdam, Rotterdam, The Netherlands; Erasmus School of Health Policy and Management, Erasmus University Rotterdam, Rotterdam, The Netherlands; Erasmus School of Health Policy and Management, Erasmus University Rotterdam, Rotterdam, The Netherlands; Erasmus School of Health Policy and Management, Erasmus University Rotterdam, Rotterdam, The Netherlands; (Social Sciences Section)

**Keywords:** Depression, Life satisfaction, Social support, Support balance quality of life

## Abstract

**Objectives:**

Although many studies have explored the benefits of support giving or receiving for older people, little is known about how the balance between giving and receiving instrumental support in nonrelative relationships affects home-dwelling older people. This study examines the relationship between long-term support balance and subjective well-being in relationships with nonrelatives among older people across 11 European countries.

**Methods:**

A total of 4,650 participants aged 60 years and older from 3 waves of the Survey of Health and Retirement in Europe were included. Support balance was calculated as the intensity difference between support received and support given across 3 waves. Multiple autoregressive analyses were conducted to test the relationship between support balance and subjective well-being, as indicated by quality of life, depression, and life satisfaction.

**Results:**

The impact of balanced versus imbalanced support on all subjective well-being measurements was not significantly different. Compared to balanced support, imbalanced receiving was negatively related to subjective well-being and imbalanced giving was not related to better subjective well-being. Compared to imbalanced receiving, imbalanced giving showed to be the more beneficial for all subjective well-being measures.

**Discussion:**

Our results highlight the beneficial role of imbalanced giving and balanced support for older people compared to imbalanced receiving. Policies and practices should prioritize creating an age-friendly environment that promotes active participation and mutual support among older people, as this may be effective to enhance their well-being.

As the population ages and healthcare expenditure increases, many countries are facing budget issues for paid care. This has led policymakers to advocate for an increased reliance on unpaid care, provided by those from individuals’ personal network, such as relatives, friends, and neighbors (Coe et al., 2020; Stall et al., 2019). However, the disproportionate burden that family caregivers experience limits the potential of unpaid support from relatives. Utilizing support resources from nonrelatives, such as neighbors and friends, has been demonstrated to facilitate aging in place and is encouraged by numerous countries (Pani-Harreman et al., 2021). Although the protective effect of receiving and giving social support on health outcomes among older people has been repeatedly proven by studies, few have investigated how the balance between giving and receiving support in relationships with nonrelatives affects health outcomes for older people (Hoogerbrugge & Burger, 2018; Lin et al., 2014; Wu & Sheng, 2019). In this article, we apply the term nonrelative to describe people who do not have a familial relationship with the one they support and/or receive support from. Support balance is conceptualized as the difference in instrumental support that older people have given and received to and from nonrelatives, and its impact on older people’s subjective well-being, as indicated by quality of life (QoL), depression, and life satisfaction, is examined based on three-waves data.

## Background

The most common categorization for measuring social support is the division of emotional support and instrumental support (Mohd et al., 2019). Compared to emotional support, which concerns the expression of emotion and the general need for companionship, instrumental support refers to tangible forms of assistance one receives or provides to serve more specific needs (Cohen & Wills, 1985). Studies suggest that emotional support and instrumental support may work differently and can bring various outcomes for individuals (Morelli et al., 2015). This study particularly focuses on instrumental support and conceptualizes it as practical help, including personal care, practical household help, or paperwork.

The relationship between support giving and receiving can be either balanced or imbalanced. Balanced support refers to relationship dynamics in which the amount of support given is equal to the amount received, while imbalanced support refers to the relationship in which the amount of support given and received is disproportionate (Fyrand, 2010). Social exchange theory and equity theory are the most commonly used theories in research on support balance. According to social exchange theory, individuals are rational decision-makers who tend to maximize their benefits as rewards and minimize cost in interpersonal relationships (Homans, 1958). Following this idea, individuals are expected to be most benefited when they receive more support than they give. Compared to that, equity theory claims that individuals would prefer to maintain a balance of exchanges and prefer relationships where the amount of support received and given is relatively equal, as support imbalance can cause feelings of distress, guilt, or overburden and negatively affect individuals (Fyrand, 2010; Hatfield et al., 1978).

Despite numerous findings supporting the beneficial role of balanced support on health outcomes, whether this relationship specifically applies to support between older people and their nonrelatives has not been thoroughly investigated. The role of support balance should be considered within the context of different relationships over time. Because of strong existing societal norms and expectations that require relatives to help each other, relationships between close relatives are less likely to be terminated even if the support reciprocity is imbalanced (Thomas, 2010). Therefore, balanced reciprocity cannot be fully applied in relationships between older people and close relatives. In contrast, instrumental support exchange with nonrelatives, such as neighbors and friends, tends to be more in line with balanced reciprocity, given that there are less strict societal norms and expectations related to support exchange, individuals are inclined to end the relationship when they feel unsatisfied with the imbalanced exchange (Li et al., 2011). Some empirical studies have supported applying equity theory in understanding support balance. Wang (2019) found that the perception of support imbalance was associated with poorer psychological well-being compared to balanced support. A 23-year follow-up study found that adults who have balanced instrumental support had a lower risk of all-cause mortality than those who had imbalanced support (Chen et al., 2021). However, these studies did not either focus on instrumental support nor specifically on relationships with nonrelatives. Based on arguments above, we propose the first hypothesis that balanced instrumental support with nonrelatives will be associated with better subjective well-being than imbalanced support, indicated by higher level of QoL, life satisfaction, and a lower level of depression (H1).

Support exchange is considered imbalanced when the giving and receiving are not equal. According to the esteem-enhancement theory, providing support to someone and being underbenefited leads to enhanced self-esteem and increased well-being. On the contrary, over-receiving support leads to negative self-evaluation and may resultantly damage health outcomes (Batson & Powell, 2003). Lack of repayment for received support may push the support recipient into a psychological state of indebtedness, threatening the individual’s sense of independence, igniting feelings of guilt, and increasing distress (Brown et al., 2003; Silverstein et al., 1996). Alternatively, providing support makes one feel independent and increases the feelings of self-esteem and mastery, which are particularly beneficial for the well-being of older people (Irby-Shasanmi & Erving, 2020). A lifespan perspective of social support suggests that the impact of support varies across different age demographics (Li et al., 2011). Younger people tend to focus more on their self-concept and development, which makes support receiving more important, while older people focus more on their contribution to society and are more willing to help others (Uchino, 2009). Importantly, for older people, providing more support than they receive to nonrelatives implies that they can still contribute to the society, which enhances feelings of confidence (Conkova et al., 2018).

Evidence related to how imbalanced support in relationships with nonrelatives affect older people is limited. Results from a study focusing on people with chronic mental health disorders suggested that providing peer support is more beneficial than receiving support (Bracke et al., 2008). Similarly, a study explored the effect of giving support versus receiving support on longevity in older married adults (Brown et al., 2003). Results revealed that providing instrumental support to friends, relatives, and neighbors reduced the mortality risk, while receiving instrumental support from others increased the mortality risk. Importantly, giving support counterbalanced the negative effect of receiving support. However, these results did not distinguish the effect of each relationship. Thomas (2010) distinguished relationship types and found similar results for older people, although this study failed to focus on instrumental support specifically. Also, these studies measured support giving and receiving separately, none of them explored this from a balanced perspective. Based on esteem-enhancement theory, this study proposes a second hypothesis that, for imbalanced support, giving in relationships with nonrelatives, will be associated with better subjective well-being compared to imbalanced receiving, as indicated by higher level of QoL, life satisfaction, and lower level of depression (H2).

## Relevance and Aim

Previous research provided limited evidence about the impact of support balance on well-being among older people. To our knowledge, existing literature has not focused on the relationship between balance of instrumental support and subjective well-being in nonrelative relationships for older people specifically. Moreover, existing findings are primarily based on studies that collected data at a single point in time. The concept of “support bank” suggests that individuals maintain a mental record of support they have received and given (Antonucci & Jackson, 1986). Support at earlier time points can be assumed to accumulate over time, similar to the accumulation of funds in a saving account, which may affect one’s health outcomes in the long term. Based on this concept, it is more suitable to measure support balance over time for a long-term perspective while discussing its impact on subjective well-being. To fill in these gaps, this study aims to focus on instrumental support and investigate the relationship between social support balance with nonrelatives and subjective well-being of older people.

## Method

### Data

Data from Survey of Health, Ageing, and Retirement of Europe (SHARE) is used in the present study (Börsch-Supan et al., 2013). SHARE is a longitudinal community-based survey conducted biannually with computer-assisted personal interviews that focuses on health, well-being, socioeconomic, and social relationships among a European population aged 50 and older and their partners. The first data collection was conducted in 2004 with respondents from 11 countries and has expanded up to 27 European Union countries and Israel in Wave 8 (2019). More detailed information of SHARE could be found at: https://share-eric.eu/.

Data of this study stem from Waves 4 (2011), 5 (2013), and 6 (2015). We chose multiple waves to test the effect of long-term support balance. In the SHARE data set, Waves 3 and 7 are outliers, because a different set of questions were asked (related to the life history of respondents), which makes Waves 4, 5, and 6 the most recent consecutive set of standard SHARE data collection. This strategy allows us to calculate cross-time balance as well as prevents inappropriate calculation that are influenced by the data variation during Waves 3 and 7 and prevents large data gaps in calculating cross-time balance. Data of the included three waves will be respectively referred to as Time 1 (T1), Time 2 (T2), and Time 3 (T3) in the present study.

**Figure 1.**
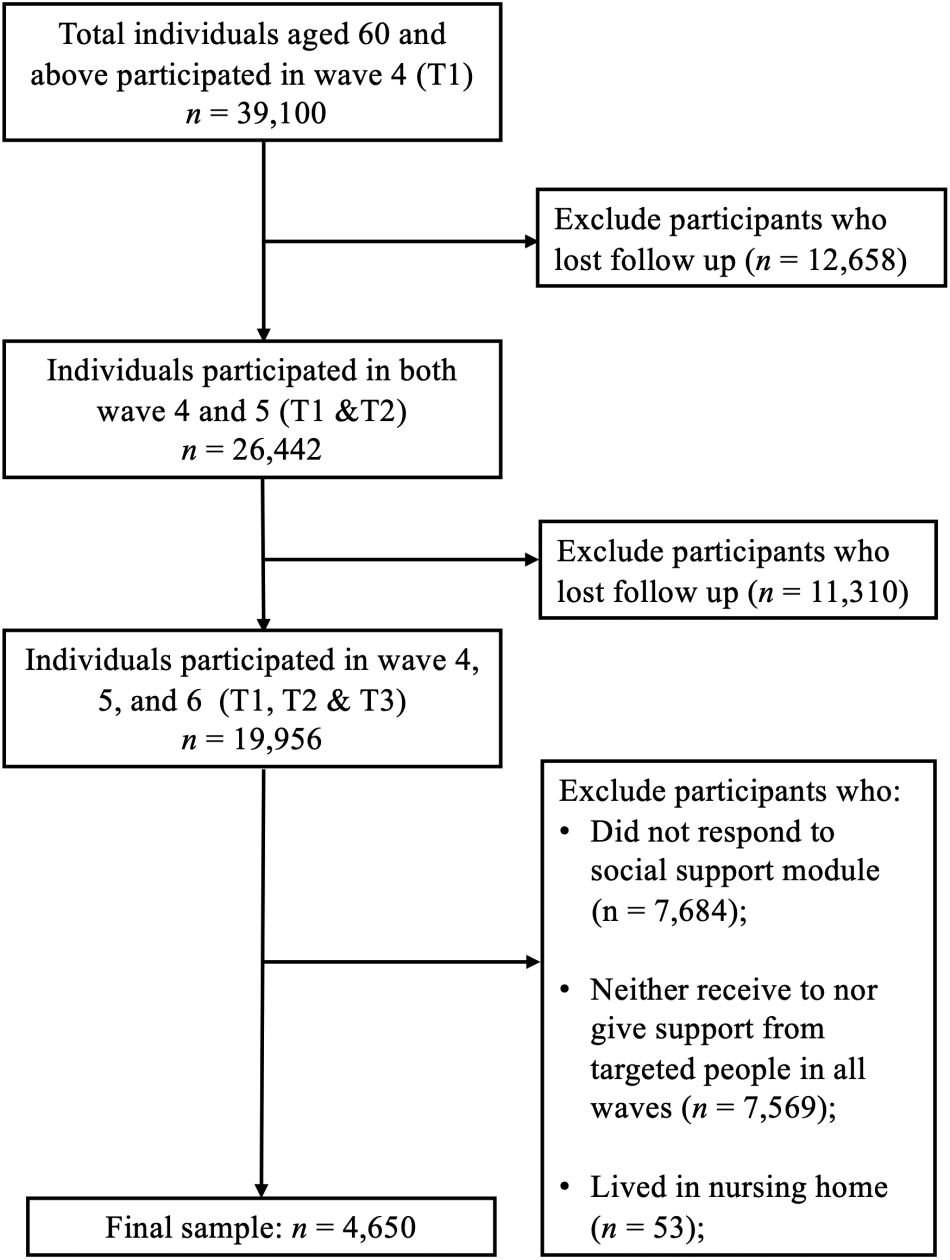
Flow chart of sample selection.

The study had five exclusion criteria. First, we excluded individuals who aged younger than 60 at Wave 4. Second, individuals who did not participate in each selected wave were excluded. Third, we excluded those who did not provide answers to the social support module, which contains the key variables of this study. Fourth, individuals who had never offered nor received any support from people outside their household in all three waves were excluded because support balance outside of the household was absent. In other words, those who had never received or given any support and those who only had support interaction with family members across all waves were excluded. Last, individuals who live in nursing homes were excluded from the sample. The sample selection process is summarized in Figure 1.

### Variables Construction

#### Independent variables

The independent variable is the long-term support balance between support that has been provided and received among older people, which is conceptually in line with previous studies (Chen et al., 2021; Irby-Shasanmi & Erving, 2020; Li et al., 2011). To get the long-term support balance indicator, we first calculated the support intensity of giving and receiving separately. Participants were asked to recall their experience of receiving and giving support to people outside the household in the past 12 months. We calculated the intensity of support received from nonrelatives as the sum frequency ranging from 0 to 12 for each wave, and the average frequency across three waves was calculated as the long-term intensity of receiving support. Similarly, support giving was calculated as the averaged frequency across three waves of the intensity of giving support to nonrelatives. Finally, support balance was calculated as the difference between the averaged intensity of giving and receiving, that is, the waves-averaged frequency of support given minus that of support received. We created two separate categorical variables to capture (im)balance. In the first categorical variable, balanced support was coded as 0 and both negative and positive imbalances were coded as 1. In the second categorical variable, negative scores were categorized as imbalanced receiving, which means more receiving than giving; 0 was categorized as balanced support, and positive scores were categorized as imbalanced giving, that is, giving more support than receiving. Variables with different categorization strategies were put into different models for analysis. The detailed calculation of independent variable is presented in Supplementary Material.

#### Dependent variables

##### Quality of life

Quality of life was assessed using the CASP-12 scale, the abridged version of CASP-19, with good psychometric properties with good reliability and validity (Borrat-Besson et al., 2015; Lestari et al., 2021). The CASP scale was developed based on the need satisfaction theory, which posits that human beings share a common set of needs, and the extent to which these needs are fulfilled reveals the level of subjective well-being of individuals (Diener & Lucas, 2000). Twelve items in the scale measure the frequency of individuals’ experienced feelings related to the four dimensions with answers ranging from “often,” “sometimes,” to “rarely” and “never,” which were coded from 1 to 4. The sum of all 12 items was calculated as the score of CASP scale, ranging from 12 to 48. A higher score indicates better subjective well-being and QoL (Ateca-Amestoy & Ugidos, 2013; Borrat-Besson et al., 2015). Cronbach’s α in the present study was 0.79 to 0.82 from T1 to T3.

##### Depression

Depressive symptoms are measured by the EURO-D scale, originally developed as a unified tool for assessing depressive symptoms across countries (Copeland et al., 2004). The EURO-D consists of 12 items evaluating the presence of 12 depressive symptoms in the last month, including depression, pessimism, death wishes, guilt, sleep, interest, irritability, appetite, fatigue, concentration, enjoyment, and tearfulness. Each item is scored 0 (symptom not present) or 1 (symptom present), and item scores are summed (0–12) as the score measuring the level of depression, a higher score refers to higher level of depression. The Cronbach’s alpha for samples in this study was 0.67 to 0.68 from T1 to T3.

##### Life satisfaction

Life satisfaction is frequently used to assess the overall well-being of individuals as it allows respondents the flexibility to weight the value of specific life domains by their own standards to assess their life satisfaction and has been shown to have adequate reliability and validity (Brandt et al., 2022). In this data set, it was measured by individuals’ responses to a single question: “On a scale from 0 to 10 where 0 means completely dissatisfied and 10 means completely satisfied, how satisfied are you with your life?” Thus, higher values indicate higher life satisfaction.

### Covariates

Control factors that have been shown in other studies to influence well-being were included as covariates, including demographic, socioeconomic (i.e., educational level, financial stress, and employment status), and health-related factors at baseline (Barbosa et al., 2020; Lestari et al., 2020; Santini et al., 2020). Age (continuous) and gender (male/female) were controlled. Marital status was grouped as “partnered” (married/registered partner) or “not partnered” (separated/never married/divorced/widowed). Employment status was dichotomized into employed and unemployed. Eleven European countries were grouped Southern (Spain, Italy), Northern (Sweden, Denmark), Western (Germany, France, Switzerland, Belgium, Austria, and Czech Republic), and Eastern (Slovenia, Estonia) European countries (Djundeva et al., 2019). Education level was coded according to the International Standard Classification of Education and was classified as lower (0–2), middle (3–4), and higher (5–6). Financial factors were studied by measuring financial stress and equivalized income. The former indicator was measured by the difficulty participant have to meet their needs, answers were coded from easy to great difficulty (1–4), and the latter was measured by dividing the gross household income by the square root of household size (Angelini et al., 2019). Health was measured by self-rated health (SRH) ranging from poor to excellent (1–5) and the number (0–6) of limitations respondents experience with activities of daily living (ADLs) and instrumental activities of daily living (IADLs). In addition, we also included the dependent variables in Wave 4 and Wave 5 in equations to build autoregressive models, given that it may provide stronger evidence for a causal relationship from a cross-time perspective (Selig & Little, 2012).

### Analysis

There are 921 observations (19.78%), which had missing values on at least one variable of interest. Little’s MCAR test showed that data were not missing completely at random (χ^2^(3684) = 5,689.96, *p* < .001). To maximize the statistical power while minimizing bias, Multivariate Imputation by Chained Equations (MICE) was conducted to compensate the missing values with the “mice” package in R. Test of multicollinearity for all variables resulted in the variance inflation factor scores ranging from 1.05 to 1.45, indicating no concerns about multicollinearity. To test the relationship between support balance and well-being indicators, multiple regression analyses controlled for covariates were conducted. In the first three regression models, imbalanced giving and imbalanced receiving were combined to produce a dummy variable with two categories including balanced support and imbalanced support, while variables of QoL, depression, and life satisfaction in T3 were set as dependent variables in each model. In the follow-up analyses, support balance was categorized as three factors of imbalanced giving, balanced support, and imbalanced receiving. In all regression models, dependent variables in T1 and T2 were included, providing a longitudinal view of the relations as well as stronger evidence of the relationships. To test the robustness of results, additional regressions without dependent variables in T1 and T2 were conducted as sensitivity analyses. All data analyses were conducted using R version 4.2.2. *p* < .05 was considered statistically significant.

## Results

### Descriptive Results

Sample characteristics are presented in Table 1 (data characteristics in each sample exclusion step are presented in Supplementary Table 1). A total of 4,650 participants with a mean age of 70.64, ranging from 60 to 97, were included in the study. As shown in Table 1, more than half of participants were female and approximately 60% of participants were from western Europe. Support between receiving and giving was imbalanced for over 95% of participants. Importantly, more than half of participants (54.86%) reported to have given more support than received, which is consistent with the support patterns found in other studies (Chen et al., 2021; Li et al., 2011).

**Table 1.** Sample Characteristics (*N* = 4,650)

Variable	Mean	*SD*	*N* (%)
Age	70.64	7.46	
Gender (female)			3,030 (65.16)
Regions			
South			341 (7.33)
North			641 (13.78)
East			836 (17.98)
West			2,832 (60.90)
Employee status (working)			460 (9.89)
Marital status (partnered)			1,856 (39.91)
Education			
Lower			1,713 (36.84)
Middle			1,830 (39.35)
Higher			1,107 (23.81)
Financial difficulty	2.13	0.96	
Equivalized income	9.67	0.96	
ADL	0.26	0.80	
IADL	0.39	0.97	
Self-rated health	2.76	1.09	
Quality of life T1 (12–48)	37.12	6.43	
Quality of life T2	37.28	6.40	
Quality of life T3	37.06	6.53	
Depression T1 (0–12)	2.73	2.27	
Depression T2	2.65	2.28	
Depression T3	2.67	2.23	
Life satisfaction T1 (0–10)	7.52	1.88	
Life satisfaction T2	7.38	1.96	
Life satisfaction T3	7.59	1.88	
Cross-time support balance			
Balanced support			213 (4.58)
Imbalanced support			
Imbalanced giving			2,551 (54.86)
Imbalanced receiving			1,886 (40.56)

*Note*: ADL = activities of daily living; IADL = instrumental activities of daily living; *SD* = standard deviation; T1 = Time 1; T2 = Time 2; T3 = Time 3.

### Relationship Between Balanced Versus Imbalanced Support and Subjective Well-Being

Table 2 shows the relationship between (im)balanced support and subjective well-being. Comparing the different effects of balanced and imbalanced support, there were no significant differences for all subjective well-being indicators. Although age was negatively related to QoL (β = −0.060, *p* < .001) and positively related to depression (β = 0.012, *p* < .001), the effect of increasing age on subjective well-being is minimal. Compared to those from Western Europe, participants from Eastern Europe had a lower level of QoL (β = −0.592, *p* < .01) and life satisfaction (β = −0.248, *p* < .001), whereas participant from Northern Europe had a higher level of life satisfaction (β = 0.217, *p* < .01). Those from southern Europe had a higher level of depression (β = 0.229, *p* < .001). Middle (β = 0.383, *p* < .05) and higher (β = 0.506, *p* < .01) levels of education were related to higher level of QoL but not related to depression and life satisfaction, compared to lower level of education. Financial stress was negatively related to lower QoL (β = −0.289, *p* < .001), life satisfaction (β = −0.142, *p* < .001), and more symptoms of depression (β = 0.070, *p* < .05). Similarly, participants with higher level of self-rated health reported a higher level of QoL (β = 0.586, *p* < .001), life satisfaction (β = 0.166, *p* < .01), and less symptoms of depression (β = −0.190, *p* < .001).

**Table 2.** Regression Results for Relationships of (Im)balanced Support and Subjective Well-Being

Variable	Quality of life T3		Depression T3		Life satisfaction T3	
	Estimate	*SE*	Estimate	*SE*	Estimate	*SE*
Imbalanced vs balanced	−0.283	0.306	0.123	0.121	−0.039	0.106
Age	−0.060^***^	0.010	0.012^***^	0.004	0.001	0.003
Gender	0.222	0.141	−0.221^***^	0.056	−0.064	0.049
North	−0.135	0.195	−0.151^*^	0.077	0.217^**^	0.067
South	−0.119	0.261	0.229^***^	0.102	−0.072	0.089
East	−0.592^**^	0.198	0.036	0.078	−0.248^***^	0.069
Employment	−0.015	0.231	−0.138	0.091	−0.001	0.080
Marital Status	0.251	0.138	0.052	0.055	0.067	0.048
Education (low)						
Middle	0.383^*^	0.155	−0.105	0.061	0.012	0.053
High	0.506^**^	0.183	−0.059	0.072	−0.027	0.063
Financial stress	−0.289^***^	0.079	0.070^*^	0.030	−0.142^***^	0.027
Equivalized income	0.160^*^	0.082	0.033	0.032	−0.001	0.028
ADL	−0.007	0.104	−0.019	0.041	−0.049	0.036
IADL	−0.217^*^	0.075	0.130	0.035	−0.084	0.031
Self-rated health	0.586^***^	0.075	−0.190^***^	0.029	0.166^**^	0.025
Quality of life T1	0.243^***^	0.015				
Quality of life T2	0.435^***^	0.014				
Depression T1			0.231^***^	0.014		
Depression T2			0.351^***^	0.014		
Life satisfaction T1					0.214^***^	0.015
Life satisfaction T3					0.293^***^	0.014
Intercept	13.483^***^	1.003	0.486	0.334	3.709^***^	0.407
Adjusted *R*^2^	0.562		0.413		0.358	

*Notes*: ADL = activities of daily living; IADL = instrumental activities of daily living; *SE* = standard error; T1 = Time 1; T2 = Time 2; T3 = Time 3.

Reference groups: gender = female; regions = west; employment = unemployed; marital status = not partnered; education level = low.

^***^ *p* < .001.

^**^ *p* < .01.

^*^ *p* < .05.

In the follow-up steps, we separated the imbalanced support into imbalanced giving and imbalanced receiving groups. We performed two regression models for each dependent variable: first model included reference group BS, and the second model included IG as reference group. Table 3 combines these two regression models (see Supplementary Tables 2 and 3 for detailed results on the separate regression models). There were no significant differences regarding the effect of imbalanced giving compared to balanced support on all well-being measures. Imbalanced receiving compared to balances support was related to a lower level of QoL (β = −0.832, *p* < .01), a higher level of depression (β = 0.286, *p* < .05), but not a significant different level of life satisfaction. Comparison between imbalanced giving and imbalanced receiving showed that imbalanced receiving was related to a lower QoL (β = −0.883, *p* < .001), lower life satisfaction (β = −0.188, *p* < .001), and more symptoms of depression (β = 0.261, *p* < .001). The relationships between demographic factors and well-being were in line with the previous regression. Similarly, participants with less financial stress and better self-rated health experienced better subjective well-being, as indicated by all measures. Additional sensitivity analysis, where dependent variables in T1 and T2 were removed from regressions, showed parallel patterns although effect sizes decreased (see Supplementary Tables 4 and 5). In general, results above showed that giving more support than receiving is not differently related to subjective well-being, while over-receiving support from others is related to worse subjective well-being.

**Table 3.** Regression Results for Relationships Between Support Balance Types and Subjective Well-Being

Variable	Quality of life T3		Depression T3		Life satisfaction T3	
	Estimate	*SE*	Estimate	*SE*	Estimate	*SE*
Imbalanced giving vs balanced support	0.051	0.310	0.025	0.122	0.109	0.107
Imbalanced receiving vs balanced support	−0.832^**^	0.318	0.286^*^	0.126	−0.079	0.110
Imbalanced receiving vs imbalanced giving	−0.883^***^	0.146	0.261^***^	0.058	−0.188^***^	0.050
Age	−0.047^***^	0.010	0.008^*^	0.004	−0.003	0.003
Gender	0.175	0.140	−0.210^***^	0.056	−0.074	0.049
Regions (West)						
North	−0.163	0.194	−0.142	0.077	0.211^**^	0.067
South	−0.132	0.260	0.228^*^	0.102	−0.070	0.089
East	−0.618^**^	0.197	0.044	0.078	−0.256^***^	0.069
Employment	0.025	0.231	−0.141	0.091	−0.001	0.080
Marital status	0.216	0.138	0.064	0.055	0.060	0.048
Education (low)						
Middle	0.373^*^	0.155	−0.102	0.061	−0.009	0.053
High	0.519^**^	0.183	−0.063^**^	0.072	−0.025	0.063
Financial stress	−0.307^**^	0.079	0.074^*^	0.030	−0.144^***^	0.027
Equivalized income	0.193^*^	0.081	0.025	0.032	0.005	0.028
ADL	0.021	0.104	−0.027	0.041	0.042	0.036
IADL	−0.154	0.090	0.111^**^	0.036	−0.070^*^	0.031
Self-rated health	0.537^***^	0.075	0.173^***^	0.029	0.154^***^	0.025
Quality of life T1	0.242^***^	0.015				
Quality of life T2	0.428^***^	0.014				
Depression T1			0.230^***^	0.014		
Depression T2			0.347^***^	0.014		
Life satisfaction T1					0.213^***^	0.015
Life satisfaction T2					0.291^***^	0.014
Adjusted *R*^2^	0.566		0.415		0.360	

*Notes*: ADL = activities of daily living; IADL = instrumental activities of daily living; *SE* = standard error; T1 = Time 1; T2 = Time 2; T3 = Time 3.

Reference groups: gender = female; regions = west; employment = unemployed; marital status = not partnered; education level = low.

^***^ *p* < .001.

^**^ *p* < .01.

^*^ *p* < .05.

**Figure 2.**
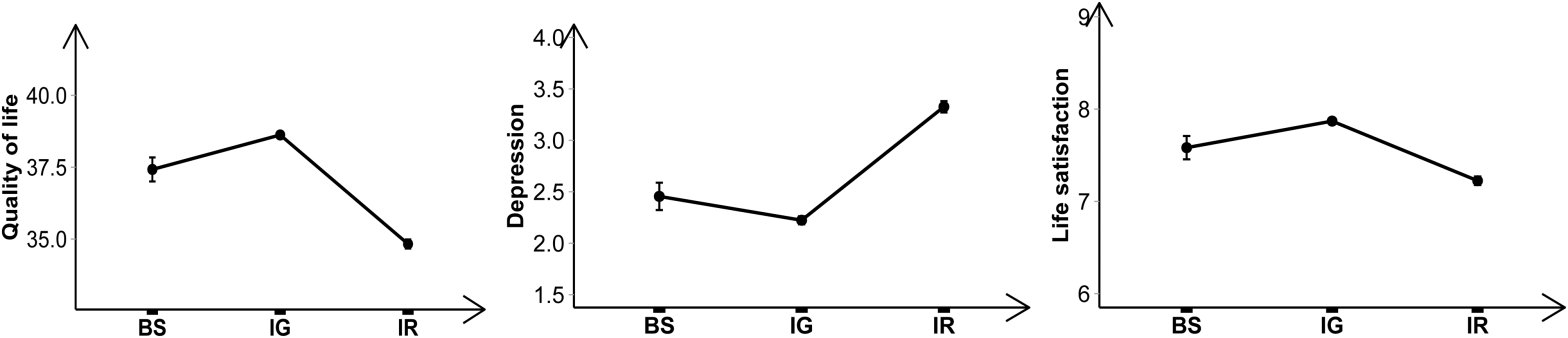
Means of subjective well-being indicators by types of support balance. BS = Balanced support; IG = Imbalanced giving; IR = Imbalanced receiving.

Figure 2 displays the means of subjective well-being indicators by types of support balance. Although the difference between balanced support and imbalanced giving was not significant, there was a trend that imbalanced giving was related to better subjective well-being in all indicators.

## Discussion

Overburden of family caregivers makes instrumental support from nonrelatives more important for older people. This study focused specifically on the relationships between older people and nonrelatives. Using a cross-time measurement of support balance, our findings indicate that balanced support or imbalanced giving with nonrelatives is related to a higher level of subjective well-being for older people than imbalanced receiving.

Previous related studies claimed that balanced support is more beneficial for older people compared to either imbalanced giving or imbalanced receiving, while our results showed that balanced support is not associated with better subjective well-being than imbalanced support, which seems to falsify our first hypothesis. Follow-up analyses showed that although imbalanced receiving is associated with a lower level of well-being than balanced support, imbalanced giving is not. Specifically, balanced support and imbalanced giving were both associated with higher level of well-being than imbalanced receiving.

Our results are not in line with the social exchange theory and partly with equity theory (Supplementary Table 6), which is different from the previous studies (Chen et al., 2021; Wang & Gruenewald, 2019). However, Wang (2019) measured the perception of balance rather than the intensity of support behavior as we did and failed to distinguish between instrumental and emotional support. Given the different roles of emotional support and instrumental support, the measurement that combines support types may counterbalance each other. In contrast with our study, Chen (2021) did not focus on relationships with nonrelatives nor on older people particularly. While the lifespan perspective of social support suggests that the effect of social support on people’s psychological well-being varies according to their age (Ingersoll-Dayton & Antonucci, 1988; Li et al., 2011). Declining physical condition and withdrawing from the labor market result in a decreased capacity to pay back what was received, which triggers feelings of indebtedness and makes older people more sensitive to the negative feeling of over-receiving than younger people. At the same time, giving support especially to nonrelatives, offers an opportunity for older people to feel that they still provide community value and a sense of contribution, and thus may have protective effects on the negative effect of receiving support (Thomas, 2010).

Additionally, another possible reason why we found different results from previous studies might relate to the cross-time design of balance calculation in our study. Although previous studies measured balance at one single time point, we measured multiple points in time. The concept of “support bank” suggests that individuals keep track of the support they exchange with others. The cross-time measurement might therefore be more accurate in capturing the concept of balance.

We found a significantly better effect on subjective well-being from imbalanced giving than from imbalanced receiving, which confirmed our second hypothesis. It is worth noting that there was a trend suggesting that giving more than receiving is most beneficial to well-being, although the difference with balanced support did not reach statistical significance. This result appears to support the esteem-enhancement theory. Providing help to others might be beneficial even if there is no balanced reward because the behavior of caring for others itself is constructive and restorative. Although another study demonstrated the esteem-enhancement theory in intimate relationships (Väänänen et al., 2005), our study suggests that it may also apply to relationships with nonrelatives for older people.

In addition, the esteem-enhancement theory might explain the negative effect of imbalanced receiving. Overseeking help from others means one must admit to lacking the competence to cope independently, and thus bring negative effects to one’s self-esteem. Consequently, receiving imbalanced support may lead to distress, while giving more support enhances well-being (Liang et al., 2001). Our findings are consistent with previous finding that older people who overreceived support reported more anger than those who underreceived, because the inability to reciprocate undermines their sense of independency and self-esteem (Bracke et al., 2008).

Cautions need to be paid when explaining our findings. First, we measured self-reported support intensity to calculate support balance. However, participants may overestimate the support they have provided, as individuals tend to underreport the support they have received (Lin & Wu, 2018). The sense of balance may not always align with an equal amount of support-exchange behavior. One may perceive a relationship as balanced even when the exchange of support behavior is imbalanced (Fyrand, 2010). Therefore, the beneficial effect of imbalanced support giving might be overestimated. Second, although we performed autoregressive models, which are considered to give stronger evidence for causal relationship compared to simple regressions due to its cross-time design, this does not imply that the relationship between support balance and subjective well-being is unidirectional. Previous studies have demonstrated a reciprocal relationship between social support and health outcomes for older people (Santini et al., 2015; Schwartz & Litwin, 2019). Future research should be conducted to test the bidirectional relationship between support balance and subjective well-being with analysis methods such as cross-lagged analysis. Additionally, country variance should be noticed when explaining our results. Different social support characteristics across European countries have been repeatedly found in previous research (Courbage et al., 2020; Maia et al., 2022). How support balance with nonrelatives affects older people across different cultural contexts could be explored in future research.

Several limitations should be noted in this study. First, strict exclusion criteria for sample selection have led to a large number of samples being deleted. Participants in the final samples were slightly younger, healthier, more likely to be single, had higher education and income, and were more likely to be female. This reduced the population representativeness of study population. However, considering that older people who live alone are usually more vulnerable and in need of support, and that research shows a stronger protective effect of social connection for widowed solo individuals (Schafer et al., 2022), our study still offers important contributions. Second, given that participants answered questions of support receiving on behalf of their partner in Waves 4 and 5 and answered on behalf of themselves in Wave 6, this question variation may have led to a less accurate calculation of support balance. Third, we were not able to distinguish the effects of each sub-type of support given the available data in SHARE. Previous studies suggest that different support types may have different impact on well-being (Thomas, 2010; Tomini et al., 2016). Future longitudinal studies could be conducted focusing on different support types. Last, the intensity of support was measured as the frequency of receiving and giving, which might not be very precise to conceptualize the balance between giving and receiving. Future research with more precise measurements such as the hours of support could be employed to test the effect of support balance more precisely.

In conclusion, with a population-based sample of older persons, our study highlights the advantages of providing support and the negative effects of receiving excessive support in nonfamily relationships. These findings highlight the relevance of the esteem-enhancement theory over the social exchange theory or the equity theory when it comes to support given and received by older people. Given the vital role of social support for older individuals, the results suggest that policies and practices should prioritize creating an age-friendly environment that promotes active participation and mutual support among older people, as this may be effective to enhance their well-being.

## Supplementary Material

gbad198_suppl_Supplementary_Tables_1-6

## Data Availability

This article uses data from SHARE Waves 4, 5, and 6 (DOIs: 10.6103/SHARE. w4.710, 10.6103/SHARE.w5.710, 10.6103/SHARE.w6.710), see Börsch-Supan et al. (2013) for methodological details. The SHARE data collection has been funded by the European Commission through FP5 (QLK6-CT-2001-00360), FP6 (SHARE-I3: RII-CT-2006-062193, COMPARE: CIT5-CT-2005-028857, SHARELIFE: CIT4-CT-2006-028812), FP7 (SHARE-PREP: GA N°211909, SHARE-LEAP: GA N°227822, SHARE M4: GA N°261982), Horizon 2020 (SHARE-DEV3: GA N°676536, SERISS: GA N°654221), and by DG Employment, Social Affairs and Inclusion.
