# Supplementary material for: The Effect of Long-Term (Im)balance of Giving Versus Receiving Support With Nonrelatives on Subjective Well-Being Among Home-Dwelling Older People
Source: J Gerontol B Psychol Sci Soc Sci. 2024 Jan 3;79(4):gbad198. doi: 10.1093/geronb/gbad198 (PMC10924445; doi:10.1093/geronb/gbad198)
Supplement: gbad198_suppl_Supplementary_Tables_1-6 [file gbad198_suppl_supplementary_tables_1-6.docx]

**Online Supplementary Material**

**Calculation of independent variable**

The key independent variable in this study is the long-term support balance between support that has been provided and received among older people, which is conceptually in line with previous studies (Chen et al., 2021; Irby-Shasanmi & Erving, 2020; Li et al., 2011). To get the long-term support balance indicator, we first calculated the support intensity of giving and receiving separately.

**Support receiving** In the SHARE dataset, individuals were asked whether they have received support from people outside their household with a question of “thinking about the last 12 months, has any family member from outside the household, or any friend or neighbour given you any kind of help…?” Help from outside the household could be help with personal care, practical household help, or help with paperwork. A showcard containing activities of personal care (e.g. dressing, bathing or showering, eating, getting in or out of bed, using the toilet), practical household help (with home repairs, gardening, transportation, shopping, household chores) and paperwork (e.g. filling out forms, settling financial or legal matters) was used as assistance by showing to respondents during question asking. In wave 4 and 5, help to either respondents or their husband/wife/partner was both included by asking the question “Please look at card 27. Thinking about the last twelve months, has any family member from outside the household, any friend or neighbour given you (or your husband/wife/partner) personal care or practical household help?” It should be noticed that activities of paperwork were not asked explicitly in the question but were included in the activities on the showcard. Therefore, activities of paperwork were also included in this study. In wave 6, respondents were asked about support given to themselves with question “Please look at card 27. Thinking about the last twelve months, has any family member from outside the household, any friend or neighbour given you any kind of help listed on this card?”. Activities on showcard are the same as in wave 4 and 5. If respondents answered “yes”, they were guided to identify 3 persons on a relationship list from whom they have received help most often, in which both family members and non-family members were included. After identifying three help providers, participants were further asked how often they receive help from these three persons separately, with answers ranging from 1) almost every day, 2) almost every week, 3) almost every month, and 4) less often. We reverse-coded the frequency to a sequence with higher value indicating higher frequency. An additional value of 0 was coded if respondents had never received any support from people outside household or only identified family members as support providers. This process produced a variable of received support intensity per wave ranging: 0) never; 1) less often; 2) almost every month; 3) almost every week; 4) almost every day. Sum frequency of all three people was calculated with answers ranged from 0-12 for each participant per wave. Finally, the average frequency across three waves was calculated as the long-term intensity of receiving support from non-relatives.

**Support giving** Support giving were measured by support that respondents have personally given to others. Individuals were first asked with a question of “in the last 12 months, have you personally given any kind of help . . . to a family member from outside the household, a friend, or a neighbour?” Questions about support giving used the same showcard as questions about support receiving. Respondents who answered ‘yes’ needed to identify three persons on a relationship list and give the frequency they gave help for each of them. Similar to support receiving, values were reverse-coded and 0 was additionally coded for those who had not given support to targeted group of people in the present wave, which produced a variable per wave ranging: 0) never; 1) less often; 2) almost every month; 3) almost every week; 4) almost every day. Sum frequency of all three people was calculated with answers ranged from 0-12 for each participant per wave. We further calculated the averaged frequency across three waves as the longitudinal intensity of giving support to non-relatives.

**Support balance** Support balance was calculated as the difference between the averaged intensity of giving and receiving, that is, the waves-averaged frequency of support given minus that of support received. We created two separate categorical variables to capture (im)balance. In the first categorical variable, balanced support was coded as 0 and both negative and positive imbalance was coded as 1. In the second categorical variable, negative scores were categorized as imbalanced receiving, means more receiving than giving; 0 was categorized as balanced support, and positive scores were categorized as imbalanced giving, that is, giving more support than receiving. Variables with different categorization strategies were put into different models for analysis.

**Supplementary Table 1.**  Data characteristics for each sample selection step

| Variable | Data_T1 (N = 39,100) | | |  | Data_T123 (N = 19,837) | | |  | Data_final (N = 4,650) | | | |
| --- | --- | --- | --- | --- | --- | --- | --- | --- | --- | --- | --- | --- |
|  | Mean | SD | N (%) |  | Mean | SD | N (%) |  | Mean | SD | | N (%) |
| Age | 70.86 | 7.95 |  |  | 70.06 | 7.25 |  |  | 70.64 | 7.46 | |  |
| Gender |  |  | 21,737 (55.59) |  |  |  | 11,321 (57.07) |  |  |  | 3,030 (65.16) | |
| Regions |  |  |  |  |  |  |  |  |  |  | |  |
| South |  |  | 6,524 (11.25) |  |  |  | 3,349 (16.89) |  |  |  | | 341 (7.33) |
| North |  |  | 3,082 (5.31) |  |  |  | 2,089 (10.53) |  |  |  | | 641 (13.78) |
| East |  |  | 9,686 (16.70) |  |  |  | 4,061 (20.47) |  |  |  | | 836 (17.98) |
| West |  |  | 19,808 (34.15) |  |  |  | 10,338 (52.11) |  |  |  | | 2,832 (60.90) |
| Employment |  |  | 3,307 (8,46) |  |  |  | 2,091 (10.54) |  |  |  | | 460 (9.89) |
| Marital status |  |  | 26,375 (67.46) |  |  |  | 13,388 (67.49) |  |  |  | | 1,856 (39.91) |
| Education |  |  |  |  |  |  |  |  |  |  | |  |
| Lower |  |  | 18,114 (46.32) |  |  |  | 8,577 (43.24) |  |  |  | | 1,713 (36.84) |
| Medium |  |  | 13,320 (34.07) |  |  |  | 7,066 (35.62) |  |  |  | | 1,830 (39.35) |
| Higher |  |  | 6,782 (17.35) |  |  |  | 3,848 (19.40) |  |  |  | | 1,107 (23.81) |
| Financial distress | 2.24 | 0.97 |  |  | 2.16 | 0.95 |  |  | 2.13 | 0.96 | |  |
| Income | 9.53 | 0.95 |  |  | 9.62 | 0.94 |  |  | 9.67 | 0.96 | |  |
| ADL | 0.34 | 1.01 |  |  | 0.21 | 0.74 |  |  | 0.26 | 0.80 | |  |
| IADL | 0.53 | 1.31 |  |  | 0.33 | 0.94 |  |  | 0.39 | 0.97 | |  |
| Self-rated health | 2.62 | 1.06 |  |  | 2.76 | 1.04 |  |  | 2.76 | 1.09 | |  |
| QoL T1 | 36.73 | 6.57 |  |  | 37.42 | 6.26 |  |  | 37.10 | 6.42 | |  |
| QoL T2 |  |  |  |  | 37.52 | 6.30 |  |  | 37.27 | 6.43 | |  |
| QoL T3 |  |  |  |  | 37.21 | 6.31 |  |  | 37.03 | 6.56 | |  |
| Depression T1 | 2.65 | 2.32 |  |  | 2.44 | 2.18 |  |  | 2.72 | 2.26 | |  |
| Depression T2 |  |  |  |  | 2.45 | 2.21 |  |  | 2.64 | 2.28 | |  |
| Depression T3 |  |  |  |  | 2.50 | 2.22 |  |  | 2.68 | 2.23 | |  |
| Life satisfaction T1 | 7.58 | 1.87 |  |  | 7.74 | 1.78 |  |  | 7.52 | 1.88 | |  |
| Life satisfaction T2 |  |  |  |  | 7.50 | 1.89 |  |  | 7.38 | 1.96 | |  |
| Life satisfaction T3 |  |  |  |  | 7.72 | 1.78 |  |  | 7.59 | 1.87 | |  |

Notes. ADL = activities of daily living; IADL = instrumental activities of daily living; QoL = quality of life; T1 = Time 1; T2 = Time 2; T3 = Time 3. Data_T1: Data including participants who participated in wave 4; Data_T123: Data including participants who participated in wave 4, wave 5 and wave 6; Data_final: Final data for analysis.

**Supplementary Table 2.** Regression results for relationships between support balance types and subjective well-being (reference = Balanced support)

| Variable | Quality of Life T3 | |  | Depression T3 | |  | Life Satisfaction T3 | |
| --- | --- | --- | --- | --- | --- | --- | --- | --- |
|  | Estimate | SE |  | Estimate | SE |  | Estimate | SE |
| Imbalanced giving vs. Balanced support | 0.051 | 0.310 |  | 0.025 | 0.122 |  | 0.109 | 0.107 |
| Imbalanced receiving vs. Balanced support | -0.832^**^ | 0.318 |  | 0.286^*^ | 0.126 |  | -0.079 | 0.110 |
| Age | -0.047^***^ | 0.010 |  | 0.008^*^ | 0.004 |  | -0.003 | 0.003 |
| Gender | 0.175 | 0.140 |  | -0.210^***^ | 0.056 |  | -0.074 | 0.049 |
| Regions (West) |  |  |  |  |  |  |  |  |
| North | -0.163 | 0.194 |  | -0.142 | 0.077 |  | 0.211^**^ | 0.067 |
| South | -0.132 | 0.260 |  | 0.228^*^ | 0.102 |  | -0.070 | 0.089 |
| East | -0.618^**^ | 0.197 |  | 0.044 | 0.078 |  | -0.256^***^ | 0.069 |
| Employment | 0.025 | 0.231 |  | -0.141 | 0.091 |  | -0.001 | 0.080 |
| Marital Status | 0.216 | 0.138 |  | 0.064 | 0.055 |  | 0.060 | 0.048 |
| Education (Low) |  |  |  |  |  |  |  |  |
| Middle | 0.373* | 0.155 |  | -0.102 | 0.061 |  | -0.009 | 0.053 |
| High | 0.519^**^ | 0.183 |  | -0.063^**^ | 0.072 |  | -0.025 | 0.063 |
| Financial stress | -0.307^**^ | 0.079 |  | 0.074^*^ | 0.030 |  | -0.144^***^ | 0.027 |
| Equivalised income | 0.193^*^ | 0.081 |  | 0.025 | 0.032 |  | 0.005 | 0.028 |
| ADL | 0.021 | 0.104 |  | -0.027 | 0.041 |  | 0.042 | 0.036 |
| IADL | -0.154 | 0.090 |  | 0.111^**^ | 0.036 |  | -0.070^*^ | 0.031 |
| Self-rated health | 0.537^***^ | 0.075 |  | 0.173^***^ | 0.029 |  | 0.154^***^ | 0.025 |
| Quality of life T1 | 0.242^***^ | 0.015 |  |  |  |  |  |  |
| Quality of life T2 | 0.428^***^ | 0.014 |  |  |  |  |  |  |
| Depression T1 |  |  |  | 0.230^***^ | 0.014 |  |  |  |
| Depression T2 |  |  |  | 0.347^***^ | 0.014 |  |  |  |
| Life Satisfaction T1 |  |  |  |  |  |  | 0.213^***^ | 0.015 |
| Life Satisfaction T2 |  |  |  |  |  |  | 0.291^***^ | 0.014 |
| Intercept | 12.848^***^ | 1.249 |  | 0.572^***^ | 0.462 |  | 3.529^***^ | 0.409 |
| Adjusted R^2^ | 0.566 |  |  | 0.415 |  |  | 0.360 |  |

Notes. ADL = activities of daily living; IADL = instrumental activities of daily living; T1 = Time 1; T2 = Time 2; T3 = Time 3. Reference groups: gender = female; regions = west; employment = unemployed; marital status = not partnered; education level = low; ***p < .001; **p < .01; *p < .05.

**Supplementary Table 3.** Regression results for relationships between support balance types and subjective well-being

| Variable | Quality of Life T3 | |  | Depression T3 | |  | Life Satisfaction T3 | |
| --- | --- | --- | --- | --- | --- | --- | --- | --- |
|  | Estimate | SE |  | Estimate | SE |  | Estimate | SE |
| Imbalanced receiving vs. Balanced support | -0.832^**^ | 0.318 |  | 0.286^*^ | 0.126 |  | -0.079 | 0.110 |
| Imbalanced receiving vs. Imbalanced giving | -0.883^***^ | 0.146 |  | 0.261^***^ | 0.058 |  | -0.188^***^ | 0.050 |
| Age | -0.047^***^ | 0.010 |  | 0.008^*^ | 0.004 |  | -0.003 | 0.003 |
| Gender | 0.175 | 0.140 |  | -0.210^***^ | 0.056 |  | -0.074 | 0.049 |
| Regions (West) |  |  |  |  |  |  |  |  |
| North | -0.163 | 0.194 |  | -0.142 | 0.077 |  | 0.211^**^ | 0.067 |
| South | -0.132 | 0.260 |  | 0.228^*^ | 0.102 |  | -0.070 | 0.089 |
| East | -0.618^**^ | 0.197 |  | 0.044 | 0.078 |  | -0.256^***^ | 0.069 |
| Employment | 0.025 | 0.231 |  | -0.141 | 0.091 |  | -0.001 | 0.080 |
| Marital Status | 0.216 | 0.138 |  | 0.064 | 0.055 |  | 0.060 | 0.048 |
| Education (Low) |  |  |  |  |  |  |  |  |
| Middle | 0.373* | 0.155 |  | -0.102 | 0.061 |  | -0.009 | 0.053 |
| High | 0.519^**^ | 0.183 |  | -0.063^**^ | 0.072 |  | -0.025 | 0.063 |
| Financial stress | -0.307^**^ | 0.079 |  | 0.074^*^ | 0.030 |  | -0.144^***^ | 0.027 |
| Equivalised income | 0.193^*^ | 0.081 |  | 0.025 | 0.032 |  | 0.005 | 0.028 |
| ADL | 0.021 | 0.104 |  | -0.027 | 0.041 |  | 0.042 | 0.036 |
| IADL | -0.154 | 0.090 |  | 0.111^**^ | 0.036 |  | -0.070^*^ | 0.031 |
| Self-rated health | 0.537^***^ | 0.075 |  | 0.173^***^ | 0.029 |  | 0.154^***^ | 0.025 |
| Quality of life T1 | 0.242^***^ | 0.015 |  |  |  |  |  |  |
| Quality of life T2 | 0.428^***^ | 0.014 |  |  |  |  |  |  |
| Depression T1 |  |  |  | 0.230^***^ | 0.014 |  |  |  |
| Depression T2 |  |  |  | 0.347^***^ | 0.014 |  |  |  |
| Life Satisfaction T1 |  |  |  |  |  |  | 0.213^***^ | 0.015 |
| Life Satisfaction T2 |  |  |  |  |  |  | 0.291^***^ | 0.014 |
| Intercept | 12.899^***^ | 1.223 |  | 0.597^***^ | 0.448 |  | 3.637^***^ | 0.398 |
| Adjusted R^2^ | 0.566 |  |  | 0.415 |  |  | 0.360 |  |

Notes. ADL = activities of daily living; IADL = instrumental activities of daily living; T1 = Time 1; T2 = Time 2; T3 = Time 3. Reference groups: gender = female; regions = west; employment = unemployed; marital status = not partnered; education level = low; ***p < .001; **p < .01; *p < .05.

**Supplementary Table 4.** Sensitivity analysis: regression results for relationships of (im)balanced support and subjective well-being in sensitivity analysis

| Variable | Quality of Life T3 | |  | Depression T3 | |  | Life Satisfaction T3 | |
| --- | --- | --- | --- | --- | --- | --- | --- | --- |
|  | Estimate | SE |  | Estimate | SE |  | Estimate | SE |
| Imbalanced vs. Balanced | 0.067 | 0.374 |  | 0.082 | 0.139 |  | 0.060 | 0.117 |
| Age | -0.090^***^ | 0.012 |  | 0.018^***^ | 0.004 |  | 0.006 | 0.004 |
| Gender | 0.330 | 0.172 |  | -0.593^***^ | 0.064 |  | -0.090 | 0.054 |
| Regions (West) |  |  |  |  |  |  |  |  |
| North | 0.133 | 0.239 |  | -0.242^**^ | 0.088 |  | 0.389^***^ | 0.074 |
| South | -2.017^***^ | 0.315 |  | 0.562^***^ | 0.117 |  | -0.085 | 0.098 |
| East | -0.437 | 0.241 |  | 0.077 | 0.089 |  | -0.515^***^ | 0.075 |
| Employment | -0.040 | 0.283 |  | -0.073 | 0.105 |  | -0.007 | 0.088 |
| Marital Status | 0.425^*^ | 0.169 |  | 0.084 | 0.063 |  | 0.190^***^ | 0.053 |
| Education (Low) |  |  |  |  |  |  |  |  |
| Middle | 0.752^***^ | 0.190 |  | -0.241^***^ | 0.070 |  | 0.038 | 0.059 |
| High | 0.715^**^ | 0.224 |  | -0.193^*^ | 0.083 |  | -0.029 | 0.070 |
| Financial stress | -1.355^***^ | 0.093 |  | 0.258^***^ | 0.034 |  | -0.353^***^ | 0.029 |
| Equivalised income | 0.465^***^ | 0.099 |  | 0.054 | 0.037 |  | 0.044 | 0.031 |
| ADL | -0.226 | 0.127 |  | 0.068 | 0.047 |  | -0.075 | 0.040 |
| IADL | -0.641^***^ | 0.109 |  | 0.248^***^ | 0.040 |  | -0.125^***^ | 0.034 |
| Self-rated health | 1.883^***^ | 0.085 |  | -0.549^***^ | 0.032 |  | 0.411^***^ | 0.027 |
| Intercept | 36.275^***^ | 1.414 |  | 1.974^***^ | 0.524 |  | 6.362^***^ | 0.441 |
| Adjusted R2 | 0.344 |  |  | 0.223 |  |  | 0.213 |  |

Notes. ADL = activities of daily living; IADL = instrumental activities of daily living; T1 = Time 1; T2 = Time 2; T3 = Time 3. Reference groups: gender = female; regions = west; employment = unemployed; marital status = not partnered; education level = low; ***p < .001; **p < .01; *p < .05.

**Supplementary Table 5.** Sensitivity analysis: regression results for relationships between support balance types and subjective well-being

| Variable | Quality of Life T3 | | |  | | Depression T3 | | | |  | | Life Satisfaction T3 | |
| --- | --- | --- | --- | --- | --- | --- | --- | --- | --- | --- | --- | --- | --- |
|  | Estimate | | SE |  | | Estimate | | SE | |  | | Estimate | SE |
| Imbalanced giving vs. Balanced support | 0.668 | | 0.377 |  | | -0.084 | | 0.140 | |  | | 0.166 | 0.118 |
| Imbalanced receiving vs. Balanced support | -0.942^*^ | | 0.387 |  | | 0.361^*^ | | 0.144 | |  | | -0.119 | 0.122 |
| Imbalanced receiving vs. Imbalanced giving | -1.609^***^ | | 0.177 |  | | 0.445 | | 0.066 | |  | | -0.286 | 0.056 |
| Age | -0.067^***^ | | 0.012 |  | | 0.011^**^ | | 0.004 | |  | | 0.010^**^ | 0.004 |
| Gender | 0.242 | | 0.171 |  | | -0.569^***^ | | 0.064 | |  | | -0.106^*^ | 0.054 |
| Regions (West) |  | |  |  | |  | |  | |  | |  |  |
| North | 0.073 | | 0.237 |  | | -0.226^*^ | | 0.088 | |  | | 0.379^***^ | 0.074 |
| South | -1.995^***^ | | 0.313 |  | | 0.556^***^ | | 0.116 | |  | | -0.081 | 0.098 |
| East | -0.484^*^ | | 0.239 |  | | 0.090 | | 0.089 | |  | | -0.523^***^ | 0.075 |
| Employment | -0.022 | | 0.281 |  | | -0.078 | | 0.104 | |  | | -0.004 | 0.088 |
| Marital Status | 0.357^*^ | | 0.168 |  | | 0.103 | | 0.062 | |  | | 0.178^***^ | 0.053 |
| Education (Low) |  | |  |  | |  | |  | |  | |  |  |
| Middle | 0.725^***^ | | 0.188 |  | | -0.233^***^ | | 0.070 | |  | | 0.033 | 0.059 |
| High | 0.732^***^ | | 0.222 |  | | -0.198^*^ | | 0.083 | |  | | -0.026 | 0.070 |
| Financial stress | -1.365^***^ | | 0.092 |  | | 0.261^***^ | | 0.034 | |  | | -0.355^***^ | 0.029 |
| Equivalised income | 0.518^***^ | | 0.099 |  | | 0.039 | | 0.037 | |  | | 0.054 | 0.031 |
| ADL | -0.170 | | 0.126 |  | | 0.053 | | 0.047 | |  | | -0.065 | 0.040 |
| IADL | -0.517^***^ | | 0.109 |  | | 0.214^***^ | | 0.041 | |  | | -0.103^**^ | 0.034 |
| Self-rated health | 1.764^***^ | | 0.086 |  | | -0.516^***^ | | 0.032 | |  | | 0.389^***^ | 0.027 |
| Adjusted R^2^ | 0.356 |  | | |  | | 0.230 | |  | |  | 0.218 |  |

Notes. ADL = activities of daily living; IADL = instrumental activities of daily living; T1 = Time 1; T2 = Time 2; T3 = Time 3. Reference groups: gender = female; regions = west; employment = unemployed; marital status = not partnered; education level = low; ***p < .001; **p < .01; *p < .05.

**Supplementary Table 6.** Summary of the relationship between related theories and results

| **Theories** | **Connection with research** | **Whether theory supported** |
| --- | --- | --- |
| Equity theory | Balanced support> Imbalanced support | Partial support |
| Social exchange theory | Imbalanced receiving > (Imbalanced giving/Balanced support) | No |
| Esteem-enhancement theory | Imbalanced giving > (Imbalanced receiving/Balanced support) | Yes |
